# The three-dimensional perspective of CAR-T cell therapy in autoimmune diseases

**DOI:** 10.1016/j.ebiom.2026.106341

**Published:** 2026-06-23

**Authors:** Jiayi Shen, Jianhua Mao, Qing Ye

**Affiliations:** a Department of Nephrology, Children's Hospital, and Liangzhu Laboratory, Zhejiang University School of Medicine, Hangzhou, 310052, China; b Department of Nephrology, First Affiliated Hospital of Zhejiang Chinese Medical University (Zhejiang Provincial Hospital of Traditional Chinese Medicine), Hangzhou, Zhejiang, 310003, China; c Department of Laboratory Medicine, Children's Hospital, and Liangzhu Laboratory, Zhejiang University School of Medicine, Hangzhou, 310052, China

**Keywords:** CAR-T cell therapy, Autoimmune diseases, Immune remodelling, Targeted therapy, Precision therapy

## Abstract

Chimeric antigen receptor T (CAR-T) cell therapy has achieved remarkable success in haematological malignancies, ushering in the era of adoptive cell therapy. In recent years, CAR-T cell therapy has been progressively explored in autoimmune diseases (AIDs), with the goal of inducing deep immune remodelling and durable drug-free remission in selected patients. This review evaluates CAR-T cell therapy in AIDs through three clinically relevant dimensions: depth, breadth, and length. We use this framework to discuss therapeutic efficacy, immune remodelling mechanisms, disease spectra, target selection, long-term persistence and safety, with the aim of supporting more precise and controllable clinical development.

## Introduction

Chimeric antigen receptor T (CAR-T) cell therapy has demonstrated transformative efficacy in haematological malignancies such as B-cell lymphoma, owing to the ability of engineered T cells to specifically recognise and eliminate malignant cells.^1^ In autoimmune diseases (AIDs), CAR-T cell therapy is investigated as a way to deplete pathogenic immune cells and allow immune reconstitution. Early clinical reports and a larger case series now suggest that a single CD19 CAR-T cell infusion can induce drug-free and clinical remission across distinct systemic AIDs, including systemic lupus erythematosus (SLE), idiopathic inflammatory myositis (IIM) and systemic sclerosis (SSc).^2^, ^3^, ^4^ This landscape has also extended to neuroimmune disorders such as myasthenia gravis (MG),^5^, ^6^, ^7^ multiple sclerosis (MS),^8^ neuromyelitis optica spectrum disorder (NMOSD),^9^ autoimmune encephalitis and stiff-person syndrome (SPS).^5^^,^^10^

**Fig. 1:**
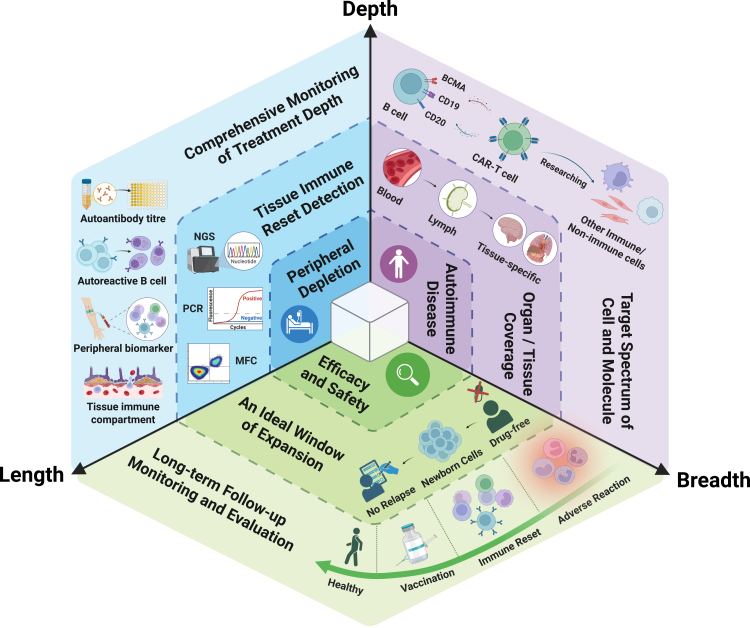
**Three-dimensional assessment of CAR-T-cell therapy for autoimmune diseases.** MFC, multiparameter flow cytometry; PCR, polymerase chain reaction; NGS, next-generation sequencing technology. Created with BioRender.com under a publication licence.

These early results need to be interpreted using endpoints that go beyond simple response or non-response. Because immune activity can persist in local compartments protected by organ barriers, CAR-T cell therapy in AIDs should be evaluated beyond peripheral blood responses. We propose three dimensions for this assessment: depth, breadth and length (Fig. 1). Depth refers to depletion of pathogenic immune compartments and tissue-level immune remodelling; breadth to the range of diseases, organs and pathogenic cell states covered; and length to drug-free remission, immune reconstitution and late toxicity rather than prolonged CAR-T detectability alone. In practical terms, these dimensions can be assessed by measurable biological indicators such as the dynamic changes of pathogenic immune cells, autoantibodies, complement levels, and some disease-specific or organ-specific readouts. These indicators are already used in clinical studies: systemic AID trials have combined disease activity scores, autoantibody and complement changes, B-cell depletion and drug-free remission; whereas neuroimmune and haematological AID reports have incorporated disease- or organ-specific measures in addition to these systemic indicators.^3^^,^^4^^,^^8^^,^^9^^,^^11^^,^^12^ We outline the current evidence to support this view and propose key challenges to guide future clinical translation.

## Depth of CAR-T cell therapy in AIDs treatment

Immune memory is essential for host defence, enabling protective immunity upon re-exposure to the same antigen, but it can also lead to relapse in AIDs. In this context, the aim of CAR-T cell therapy is not only to control symptoms, but also to deplete pathogenic immune memory.^13^ Thus, “Depth” is best defined as the extent to which CAR-T cell therapy depletes pathogenic immune cell clones and enables non-autoreactive immune reconstitution.

### Efficacy evaluation and clinical manifestations

Currently, CAR-T cell therapy in AIDs treatment primarily targets B cells or plasma cells, with efficacy assessment conducted on the basis of clinical monitoring and comprehensive evaluation of various B cell indicators. Beyond symptoms, clinical monitoring includes tissue-specific serological markers, autoantibody titres and imaging studies. In the study of CD19-targeted CAR-T cell therapy for systemic lupus erythematosus (SLE), many patients have experienced rapid alleviation of clinical manifestations, meeting the DORIS remission criteria.^2^, ^3^, ^4^ This remission is also accompanied by the disappearance of related antibodies (anti-dsDNA antibodies, nucleosome antibodies, and Smith antigen antibodies) and the normalisation of complement levels. Similar remissions have been observed in other AIDs, such as idiopathic inflammatory myositis (IIM) and systemic sclerosis (SSc),^4^^,^^14^ where patients were able to discontinue immunosuppressive drugs, including glucocorticoids. Furthermore, tracking residual B cells reveals that peripheral CD19+ B cells typically drop to undetectable levels within approximately one week of CAR-T cell infusion.^4^ This state can persist for months or even more than one year.

In neuroimmune disorders, the key question is not only whether circulating pathogenic B cells are cleared, but also whether immune activity can be altered in target organs. Because the blood–brain or blood-nerve barrier can sequester disease activity within the central nervous system (CNS) or peripheral nerve compartments, clinical improvement cannot be judged only by dynamic changes in peripheral blood.^15^ Early experience in MS indicates that organ-level readouts such as neurophysiological measures, magnetic resonance imaging (MRI) and immunoglobulin in cerebrospinal fluid (CSF) may be more informative than blood biomarkers alone.^8^ Therefore, assessment of “depth” should integrate systemic and organ-specific endpoints to fully reflect efficacy in neuroimmune disorders.

### Immune remodelling mechanism

After CAR T cell-mediated depletion, immune remodelling is usually characterised by the regeneration of predominantly naive B cells, with reduced memory B cells, plasmablasts and plasma cells.^4^^,^^16^ The absence of autoantibody rebound or clinical relapse after B-cell return is consistent with, although does not prove, restored self-tolerance. The effect on pre-existing humoural immunity depends on the selected B-cell or plasma-cell target. After CD19-targeted CAR-T cell therapy, protective vaccine immunoglobulin G (IgG) titres may be maintained because long-lived plasma cells generally lack CD19 expression.^2^^,^^6^^,^^17^^,^^18^

Data from neuroimmune disorders suggest that immune remodelling extends beyond depletion of circulating B cells and autoantibodies. CAR-T cells were detectable in the CSF without clinical signs of early neurotoxicity, and intrathecal antibody production decreased after infusion, supporting a direct effect of CAR-T cells in CNS.^8^ A recent study of anti-BCMA CAR-T therapy in progressive MS also found that CNS plasma-cell populations were depleted and the activation of microglia was reduced.^19^ Together with molecular profiling showing changes in monocyte and T-cell inflammatory programmes after CD19 CAR-T-cell therapy, these findings suggest that remodelling may involve coordinated changes in local inflammatory pathways rather than antibody depletion alone.^20^

However, the notion of complete immune remodelling should still be interpreted cautiously. It remains unclear to what extent CAR-T cell therapy durably reshapes autoreactive B-cell and T-cell repertoires, whether tissue-resident immune cells in lymph nodes and target organs are fully eradicated, and which mechanisms underlie relapse once B cells re-emerge. Future studies integrating serial B-cell and T-cell receptor repertoire sequencing, tissue sampling where feasible, and relapse-associated immune phenotyping will be necessary to distinguish true clonal deletion from temporary reconfiguration of inflammatory networks.

### Treatment depth monitoring

Studies suggest that clinical improvement is often accompanied by reductions in pathogenic autoantibodies, but this relationship is not uniform across diseases and compartments. In neuroimmune disorders, clinical recovery may precede, only partly correlate with, or even occur independently of changes in circulating autoantibody titres, indicating that target-organ immune remodelling cannot be inferred from serum measurements alone.^21^^,^^22^ The burden of autoreactive B cells is likely associated with relapse risk and the duration of remission.^23^ CAR-T cell therapy mediates deep depletion of B cells not only aids in eliminating plasmablasts that produce autoantibodies but also regulates inflammation by reducing cytokine production and abolishing the antigen presentation functions of B cells.^24^ Therefore, while using highly sensitive detection methods to quantify the therapeutic effect, it is necessary to combine peripheral immunological biomarkers with tissue- or compartment-specific readouts, especially when the disease is driven by compartmentalised inflammation behind the blood–brain or blood-nerve barriers. At present, the AIDs field lacks unified standards. Considering endpoint measures used in haematological malignancies,^25^^,^^26^ we propose adapting the minimal residual disease (MRD)^27^, ^28^, ^29^, ^30^ concept into “functional MRD” for AIDs to fully consider the immunomodulatory potential of residual cells. Specifically, depth could be monitored by assessing CD19+ B-cell depletion via multi-parameter flow cytometry (MFC), tracking the persistence of autoreactive B-cell clones through BCR sequencing, and measuring disease- or organ-specific indicators such as anti-dsDNA antibodies in rheumatological AIDs, CSF immunoglobulin or MRI activity in neuroimmune disorders, and haemolysis markers in haematological AIDs. Monitoring the dynamic changes in autoreactive B cells and assessing the function of residual cells by next-generation sequencing technology may further predict the disease activity (Table 1).

**Table 1:** Assessment for functional MRD in autoimmune disease.

Method	Sensitivity	Advantage	Limitation	Representative clinical indicators
MFC^27^	10^− 3^–10^− 4^	1 Fast 2 Low cost 3 Multiple cell subsets and immune reconstitution status	1 Fresh sample 2 Standardisation 3 Common phenotypes rather than antigen specificity	1 CD19+ B-cell depletion and reconstitution 2 CSF immune-cell changes in neuroimmune diseases 3 CAR-T cell persistence
PCR^28^	10^− 4^–10^− 6^	1 High sensitivity 2 Quantification 3 Frozen samples	1 Known sequences 2 Difficult to cover multiclonal profiles	1 CAR transgene copy number 2 CAR-T cell expansion and contraction kinetics 3 Monitoring of known pathogenic clones
NGS^29^^,^^30^	10^− 5^–10^− 6^	1 High sensitivity 2 Multi-sequence 3 Single cell level	1 High cost 2 Complex analysis 3 Poor consistency between tissue samples and blood	1 BCR/TCR repertoire diversity 2 Disappearance of pathogenic clones or relapse-associated clonal expansion 3 Paired blood–tissue repertoire comparison

MRD, Minimal Residual Disease; MFC, multi-parameter flow cytometry; PCR, polymerase chain reaction, including quantitative real-time PCR (qPCR)/digital PCR (dPCR); NGS, next-generation sequencing; CSF, cerebrospinal fluid; BCR/TCR, B-cell receptor/T-cell receptor.

## Breadth of CAR-T cell therapy in AIDs treatment

Breadth describes whether the CAR-T cell therapy reaches the disease-relevant compartments and target-cell populations that drive an autoimmune phenotype.

### Disease coverage

According to the scope of tissue involvement, AIDs can be broadly categorised into systemic and organ-specific types. Systemic AIDs exhibit immunological features such as abnormal B cell activation and the production of various autoantibodies, leading to the deposition of immune complexes in multiple organs throughout the body, accompanied by the dysregulation of T cell subsets and cytokine secretion. As previously mentioned, CD19-targeted CAR-T cell therapy has demonstrated significant efficacy in patients with systemic AIDs. It not only reduces antibody levels in the blood but also profoundly affects immune cells within lymphoid tissues, thereby altering the trajectory of long-term immune reconstitution. One study compared patients treated with CD19-targeted CAR-T cells with those treated with the CD20-targeted monoclonal antibody rituximab (RTX).^31^ After CAR-T cell infusion, CD19+ and CD20+ B cells were eliminated from lymph nodes, follicular structures were disrupted, follicular dendritic cells and follicular helper T cells were markedly reduced, and germinal centre proliferation decreased. These effects are not observed with RTX treatment. This indicates that CAR-T cell therapy can not only clear B cells but also induce complete remodelling of the lymph node structure. The disease coverage also includes haematological AIDs, in which pathogenic humoural immunity directly targets blood cells or coagulation factors, as illustrated by emerging experiences of CAR-T cell in immune thrombocytopaenia (ITP), autoimmune haemolytic anaemia (AIHA) and acquired haemophilia A.^11^^,^^12^^,^^32^

In tissue-specific AIDs, kidneys, joints, skin and CNS may all become sites of pathological immune responses, manifesting as local inflammatory damage, immune complex deposition, and persistent exposure to tissue-specific antigens. In lupus nephritis (LN), B cells abnormally infiltrate into renal interstitium and circulate through secondary lymphoid structures to collectively promote autoantibody production and drive disease ^33^; type 1 diabetes mellitus (T1DM) arises from autoreactive T cells’ attack on pancreatic β cells, accompanied by B cell infiltration and autoantibody production.^34^ Analysis of non-lymphoid tissues from the patients treated with CD19-targeted CAR-T cells revealed a complete absence of B cells (consistent with lymphoid tissue biopsy results) in the colon, kidney and gallbladder tissues after treatment, while T cells and macrophages remained present.^31^ Emerging reports in progressive MS show that CAR-T cells can reach CNS-related compartments with biological activity and are associated with reduced intrathecal antibody production.^8^^,^^19^ Clinical activity has also been reported in MG, NMOSD, SPS and autoimmune encephalitis, suggesting that CAR-T cell therapy may extend across disorders involving the neuromuscular junction, opticospinal axis, and central nervous system parenchyma.^6^^,^^9^^,^^10^^,^^35^ Together, these findings suggest that CAR-T cell therapy can achieve selective and deep immune-cell depletion across lymphoid and non-lymphoid compartments, supporting a therapeutic strategy that combines cross-system coverage with local microenvironmental modulation.

### Target selection

Target selection determines which pathogenic cell populations can be depleted and which components of protective immunity may be preserved. Initial explorations focused on B cells, with CD19 and BCMA becoming the most extensively studied antigens to date.^4^^,^^9^^,^^36^^,^^37^ CD19 remains the preferred target because it covers most B-cell states upstream of plasma cell differentiation both in peripheral circulation and tissues, and currently has the strongest clinical evidence for drug-free remission.^4^^,^^18^^,^^38^ By contrast, CD20-directed therapies such as RTX may not fully disrupt lymphoid tissue structure or deplete plasmablasts and plasma cells.^31^ BCMA-directed strategies may be advantageous when long-lived plasmablasts or plasma cells dominate the disease.^9^^,^^19^^,^^21^ Dual-target or sequential approaches may therefore be attractive when both upstream B-cell depletion and downstream plasma cell eradication are desired, though at the cost of potentially greater effects on humoural immunity. Similarly, CD3 × CD19 T-cell engagers provide a non-cellular route to deep B-cell depletion and may represent a bridging or alternative strategy in selected patients, though current evidence remains limited.^39^

Taken together, the potential value of CAR-T cell therapy over existing B-cell targeting strategies lies in its ability to induce deeper tissue depletion, disrupt pathological lymphoid structure and create the possibility of drug-free remission after a single intervention. At the same time, these potential advantages need to be balanced against lymphoid depletion, manufacturing time, inpatient monitoring and cost. Accordingly, CAR-T cell therapy should currently be viewed as a high-intensity option for selected severe or refractory patients rather than a universal replacement for established B-cell-directed agents.

Complete B cell strategies may maximise breadth across B-cell states, but at the cost of transient hypogammaglobulinaemia risks. Therefore, using CAR-T cell therapy to eliminate autoreactive antigen-specific cells represents a more precise treatment approach. Some patients with myasthenia gravis have autoantibodies against muscle-specific receptor tyrosine kinase (MuSK). Researchers have generated MuSK chimeric autoantibody receptors (CAAR) T cells and successfully reduced anti-MuSK IgG levels in mouse models without reducing total B-cell numbers or total IgG concentrations, supporting selective depletion of MuSK-specific B cells.^40^ Similarly, desmoglein 3 (Dsg3)-targeted CAAR-T cell therapy in patients with pemphigus has also shown comparable effects.^41^

Although research on T-cell relevant targets in AIDs is still in its early stage, a preclinical study by WHITTINGTON et al. demonstrated that type II collagen (CII) specific CAR-T cells significantly reduce autoimmune CD4+ T cell activation and autoantibody levels in rheumatoid arthritis (RA) mice, alleviate joint damage and delay disease onset.^42^ Additionally, FISHMAN et al. reported that targeting insulin-reactive T cells reduced the incidence of T1DM in mice,^43^ which may further expand the scope of CAR-T cell applications. In addition to B and T cells, other immune cells and certain non-immune cells also play critical roles in immune regulation, tissue inflammation and fibrosis. Fibroblast activation protein (FAP), a membrane protein linked to fibrosis, has been shown to be effectively targeted by CAR-T cells, directly reducing the fibrotic burden and even reversing the progression of tissue fibrosis.^36^

### Target exploration and breadth assessment

Target selection in AIDs requires a balance between target specificity, treatment breadth and safety (Table 2). Targeting CD19 can efficiently induce B-cell depletion and achieve deep remission in diseases such as SLE; however, the therapeutic goal in AIDs is not indefinite CAR-T cell persistence. Rather, a time-limited but sufficiently deep depletion of pathogenic B-cell compartments may be enough to permit immune reconstitution and durable drug-free remission.^38^ By contrast, BCMA-targeted CAR-T cell therapy may be particularly attractive when long-lived plasmablasts or plasma cells are dominant drivers of disease, but the objective in AIDs is controlled immune reset rather than sustained on-target pressure analogous to haematological malignancies. Therefore, target selection should balance lineage coverage, preservation of protective humoural immunity, and the desired pace of immune reconstitution.

**Table 2:** Selection of target antigens in AID for CAR-T therapy.

Target	Main Coverage	Treatment Depth and Breadth	Efficacy	Potential Risk
CD19^2^, ^3^, ^4^^,^^14^^,^^38^^,^^44^	B cells except plasma cells	1 Achieve complete elimination of B cells in peripheral blood 2 Applicable to B cell-mediated autoimmune diseases	Drug-free and clinical remission in SLE/ASS/SSc	1 Relapse by residual long-lived plasma cells 2 Mostly low-grade CRS, ICANS, transient cytopenia, infection risk, and hypogammaglobulinaemia
BCMA^45^	Memory B cells, plasmablasts and plasma cells	1 Targets long-lived plasma cells contributing to persistent autoantibody production 2 Applicable to B cell-mediated autoimmune diseases	Drug-free remission in SLE/NMOSD; clinical remission in MG	1 Protective humoural immunity may be impaired 2 CAR-T cell related toxicities, especially CRS and haematological toxicity
CD70^46^	Activated B cells and plasma cells	Eliminate the cells that produce autoantibodies	Unknown	1 Relapse by residual early-stage B cells 2 CAR-T cell related toxicities
CD38^46^	Plasmablasts and plasma cells			
CD138^46^	Plasma cells			
Antigen-specific (Dsg3/MuSK/NMDA CAAR-T)^40^^,^^47^^,^^48^	Antigen-specific autoreactive B cells	1 Precisely target pathogenic clones and retain normal immunity 2 Applicable to autoimmune diseases with known antigen	Ongoing clinical trials	1 Heterogeneity of antigenic epitopes 2 CAR-T cell related toxicities

AID, autoimmune disease; SLE, systemic lupus erythematosus; ASS, anti-synthetase syndrome; SSc, systemic sclerosis; NMOSD, neuromyelitis optica spectrum disorder; MG, myasthenia gravis; CRS, cytokine release syndrome; ICANS, Immune effector cell-associated neurotoxicity syndrome.

In addition, an ideal target should exhibit high or relative specificity within pathogenic cell lineages, enable rapid and substantial reduction of pathogenic cell populations and effective immune reconstitution (e.g. CAAR-T cell). The stability and heterogeneity of antigen expression should be evaluated before clinical use, because an overly broad target may increase immunosuppression whereas an overly narrow target may miss pathogenic clones. The differences between targets determine the potential therapeutic depth and the relapse risk. Given the differences of patients and immune microenvironments, multi-target or combination strategies are often required to achieve deeper clearance and more durable remission. Target selection should therefore strike a balance among broad coverage, precise targeting and controllable immune reconstitution, with continuous optimisation based on clinical evidence.

**Fig. 2:**
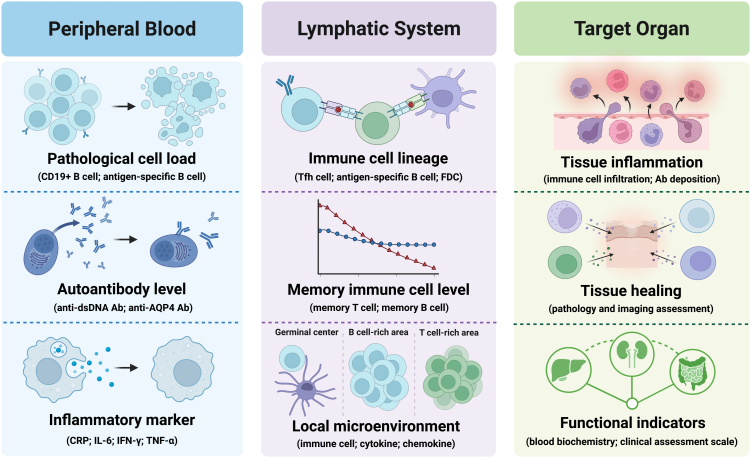
**Evaluation for the therapeutic scope of CAR-T cell therapy.** The assessment of treatment breadth mainly includes three parts: peripheral blood, lymphatic system and target organs. Some representative measurable endpoints for autoimmune diseases are shown in the figure. Ab, antibody; anti-dsDNA Ab, anti-double-stranded DNA antibody; anti-AQP4 Ab, anti-aquaporin-4 antibody; CRP, C-reactive protein; IL-6, interleukin-6; IFN-γ, interferon-γ; TNF-α, tumour necrosis factor-α; Tfh cell, T follicular helper cell; FDC, follicular dendritic cell. Created with BioRender.com under a publication licence.

Post-treatment monitoring and evaluation of CAR-T cell therapy should encompass three dynamic levels (Fig. 2): the peripheral blood level, including pathogenic cell burden, autoantibody titres and inflammatory biomarkers; the lymphoid tissue level, assessing immune cell lineage distribution, memory cell reservoirs and local inflammatory microenvironments; and the solid organ level, evaluating tissue inflammation, structural damage, repair processes, and functional outcomes. Multi-modal assessment provides a comprehensive view of disease activity across different compartments and supports dynamic optimisation of therapeutic strategies. Clinical studies should strengthen cross-level quantitative analysis and long-term follow-up to elucidate how clearance breadth influences overall disease outcomes.

## Length of CAR-T cell therapy in AID treatment

In AIDs, “length” must integrate two endpoints: sustained drug-free remission and the toxicity risk under a gene-modified cellular product.

### Durability

Unlike haematological malignancies, AIDs do not necessarily require sustained CAR-T cell persistence after initial elimination of pathogenic immune cells. Current clinical data support a model in which a transient phase of CAR-T cell expansion and B cell depletion can be followed by immune reconstitution with a re-emerging, predominantly naive B cell repertoire and continued drug-free remission.^38^ Comparative data indicate that, despite similar peak of CAR-T cell expansion, patients with SLE show shorter persistence of CAR-T cells and faster reconstitution of conventional T cells and B cells than patients with B-cell lymphoma, while also experiencing lower rates of adverse reactions.^44^ These findings suggest that prolonged persistence is not a prerequisite for efficacy in AIDs and may even be undesirable if it delays immune recovery or increases the risk of hypogammaglobulinaemia, infection or other late toxicities. Therefore, durability in AIDs should be assessed primarily by the stability of drug-free remission, the quality of immune reconstitution and the absence of relapse, rather than by prolonged detectability of CAR-T cells alone.

### Safety

The key safety concerns associated with CAR-T cell therapy in AIDs include cytokine release syndrome (CRS), immune effector cell-associated neurotoxicity syndrome (ICANS), transient cytopenia, infection risk and hypogammaglobulinaemia. Current data suggest a more favourable early safety profile than in haematological malignancies, possibly owing to lower target-cell burden and differences in disease biology.^44^ In the phase 1/2a CASTLE basket trial in severe SLE, SSc and IIM, no CRS above grade 2 and no ICANS were observed.^38^ This is consistent with long-term follow-up data from other autoimmune cohorts.^4^

Early clinical experience with CAR-T cell therapy in neuroimmune disorders does not indicate increased neurotoxicity. In progressive MS, CAR-T cells were detected in the CSF, supporting CNS compartment access without overt neurotoxicity.^19^ A study of BCMA/CD19 bispecific CAR-T cells in refractory MG reported good overall tolerability without dose-limiting toxicity or ICANS.^49^ By contrast, though no serious infections occurred, grade 2 cytokine release syndrome and moderate leucopenia were reported in advanced SPS treated with anti-CD19 CAR-T cells.^5^ These data suggest that neurological involvement itself does not inevitably confer a higher risk of ICANS. Even if early autoimmune cohorts show mostly low-grade CRS and rare neurotoxicity, the field cannot infer late risk from short follow-up, particularly when moving into patients with multi-organ vulnerability. In patients with multi-organ involvement, even low-grade toxicities may complicate organ function and clinical management.^50^ Therefore, rigorous monitoring and preventive measures should be implemented in advance.

Safety data in haematological AIDs remain limited but are encouraging. In patients with refractory SLE-associated ITP, reported treatment-related events were mainly grade 1 CRS, transient cytopenias, decreased IgG, and upper respiratory infections.^11^ In acquired haemophilia A, anti-CD19 CAR-T cell therapy achieved remission after rapid B-cell depletion and was described as safe.^12^ Likewise, a recent case of combined AIHA, ITP, and antiphospholipid syndrome was reported without CRS or ICANS.^32^ These early observations suggest that, in haematological AIDs, the dominant safety concerns are likely to be transient haematological toxicity, infection risk, bleeding-related vulnerability and hypogammaglobulinaemia rather than the high-grade immune effector toxicities.

### Follow-up and monitoring

**Fig. 3:**
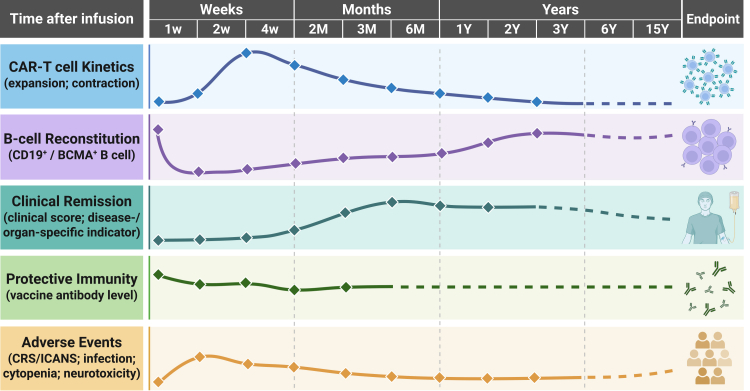
**Long-term follow-up monitoring after CAR-T cell therapy for autoimmune diseases.** The timeline highlights representative longitudinal readouts used in clinical follow-up after CAR-T cell infusion, including CAR-T cell expansion and contraction, B-cell depletion and reconstitution, clinical remission, protective immunity and adverse events. Created with BioRender.com under a publication licence.

Comprehensive and long-term follow-up data are fundamental for evaluating both the durability and safety of CAR-T therapy. The U.S. Food and Drug Administration (FDA) has recommended that all patients receiving cellular therapies undergo up to 15 years of follow-up and lifelong monitoring.^51^ Post-treatment surveillance of CAR-T cell therapy in AIDs should integrate immune reconstitution, adverse reactions and pre-existing humoural immunity in line with emerging expert recommendations.^52^ Results in a serum profiling study of patients with refractory SLE suggest that CD19-targeted CAR-T cells may only have a marginal impact on pre-existing humoural immune—profoundly reduced SLE-associated autoantibodies and inflammatory cytokines while largely preserving pre-existing humoural responses.^53^ In addition, anti-CAR immune responses should be considered when relapse occurs or repeat infusion is planned. A patient with IIM received reinfusion of the same CD19-targeted CAR-T cell product when the disease relapsed, but the CAR-T cells failed to expand and T cells targeting the CAR were detected, indicating that anti-CAR cellular immunity can impair engraftment and contribute to therapeutic failure.^54^ For neuroimmune disorders, follow-up may additionally need to include CSF studies, MRI activity and standardised neurological disability measures where appropriate. In haematological AIDs, serial monitoring of platelet counts, haemolysis biomarkers, factor VIII activity and immunoglobulin recovery should be integrated with B-cell reconstitution to capture both efficacy and delayed toxicity. These examples indicate that “length” should be judged by a composite follow-up strategy that captures remission durability, immune reconstitution, disease-specific indicators, preservation of protective immunity and adverse events (Fig. 3).

Vaccination remains a critical strategy for infection prevention, but its interpretation in AIDs should be integrated with disease-specific humoural monitoring. In haematological malignancies, one study evaluating humoural immune responses to vaccination after CAR-T therapy showed significant baseline differences in serum protection rates between CD19 and BCMA CAR-T groups, although vaccine response rates did not differ significantly.^55^ Notably, serum immunoglobulin A (IgA) levels were strongly associated with vaccine responsiveness.^55^ As IgA is primarily produced by class-switched plasma cells, the effective class switching from immunoglobulin M (IgM) to IgG/IgA reflects both presence and functional maturation of B cells. Follow-up data in AIDs similarly indicate that newly emerging B cells after CAR-T cell therapy exhibit robust vaccine responses, suggesting preserved immune resilience to antigenic challenge.^4^

## Future prospects and challenges

### Technical optimisation

To enhance the potential of CAR-T cell therapy in AIDs, multiple innovative strategies have been explored, encompassing CAR structural design, cellular sources, and manufacturing processes. Structural optimisation aims to improve target specificity and functional performance, including the development of multi-targeted CAR-T cells,^56^ logic-gated CARs,^57^ chimeric autoantibody receptors (CAARs), reduction of antigen affinity,^58^ modulation of hinge domain length,^59^ diversification of intracellular CD3 signalling domains,^60^ and incorporation of molecular safety switches.^61^ Despite these advances, autologous CAR-T cell production remains costly and time-consuming. Universal CAR-T cells derived from healthy donors offer a potential solution by shortening manufacturing time, reducing costs, and improving accessibility. Early clinical trials in AIDs have demonstrated favourable efficacy and safety profiles.^62^

However, host immune rejection limits their application. An alternative approach involves induced pluripotent stem cell (iPSC), which may reduce rejection risk.^63^ An iPSC-derived CD19/BCMA dual-target CAR-natural killer (CAR-NK) product has successfully induced substantial B-cell depletion with minimal toxicity in a patient with severe refractory SSc, supporting iPSC-derived CAR-T cells as a potentially safer and more standardised alternative for selected autoimmune indications.^64^ Another emerging strategy is using targeted lipid nanoparticles (tLNPs) that deliver CAR-encoding mRNA directly to defined T-cell subsets for in vivo CAR-T cell generation.^65^ In preclinical studies, these tLNPs reprogrammed CD8 T cells both from healthy donors and autoimmune patient samples and achieved in vivo B-cell depletion in non-human primates.^66^

Beyond CAR-T cells, CAR-NK cells (mentioned above) and CAR-engineered regulatory T cells (CAR-Tregs) offer additional therapeutic avenues. CAR-NK cells, which do not require antigen-specific priming,^67^ offer advantages including ease of procurement, uniform quality, and reduced toxicity.^68^ Although clinical experience with CAR-NK cells remains limited, further enhancement of tissue infiltration and persistence, along with combination strategies such as immune checkpoint inhibition drugs, may enable more durable and safer cellular therapies. CAR-Tregs exert immunosuppressive effects and have demonstrated efficacy with fewer adverse events in AIDs such as type 1 diabetes, ulcerative colitis, and multiple sclerosis.^69^, ^70^, ^71^ A recent preclinical study further showed that CD19-targeted CAR-Tregs restored immune homoeostasis in a humanised mouse model of SLE, restricted autoantibody generation, and protected target organs without detectable toxicity. This work highlights that future engineered cell therapy in autoimmunity might rely not only on pathogenic cell depletion but also on active restoration of immune tolerance.^72^

### Clinical challenges

Autoimmune diseases exhibit profound pathogenic heterogeneity. Even within the same disease entity, patients may differ markedly in immune drivers, infiltrating cell populations, cytokine signalling pathways, and target antigen expression profiles. Although CAR-T therapy can be highly targeted, its clinical value depends on three critical decisions: selecting appropriate patients, identifying optimal disease targets, and choosing the most suitable CAR-T product. Patient selection should be informed by emerging multidisciplinary consensus efforts, comprehensively considering disease severity, pattern of organ involvement, comorbidities, refractoriness to therapies and feasibility of structured follow-up.^52^ At present, CAR-T cell therapy is prioritised for severe or refractory AIDs after failure of established biologic or other B-cell-directed strategies, rather than routine use in earlier-line or non-life-threatening settings.

Traditional clinical trial endpoints in AIDs, such as disease activity scores, primarily reflect clinical manifestations and often lag behind underlying pathophysiological changes. CAR-T cell therapy is intended to promote deep immune remodelling, meaning that in addition to symptom improvement, long-term reduction or functional reshaping of pathogenic immune cell populations, normalisation of the immune microenvironment, and regulation of disease relevant pathways are also important. The depth–breadth–length framework can help structure these assessments. Depth may be quantified by serial CD19+ or BCMA+ B cell depletion, autoreactive clone tracking, autoantibody titres and organ-specific measures. Breadth may be assessed by whether these effects extend across peripheral blood, lymphoid tissue and target organs, or across relevant B-cell, plasma-cell and antigen-specific clones. Length could be evaluated by the duration of immunosuppressant drug withdrawal, the relapse-free survival, the kinetics of B-cell recovery, and the occurrence of adverse reactions.

The high cost associated with CAR-T cell development, manufacturing, clinical application, and long-term follow-up remains a major barrier to widespread use in AIDs. In chronic diseases, onset time, specialised inpatient monitoring, bridge therapy and long-term surveillance are likely to influence access and cost-effectiveness.^52^ Advancing universal CAR-T technologies towards scalable and standardised production may substantially reduce costs. Future studies should compare CAR-T cell therapy with standard care not only for efficacy, but also for cost-adjusted value and real-world feasibility. Moreover, continuing to conduct long-term follow-up studies, explore new clinical indications and therapeutic targets, obtain comprehensive clinical evidence and economic evaluations will be critical for facilitating broader clinical translation.

## Conclusion and perspectives

By selectively eliminating pathogenic immune cells, CAR-T cell therapy may shift therapeutic goals in AIDs from symptom control towards deeper immune intervention with the potential for durable remission in selected patients. Depth depends on precise intervention in disease mechanisms, breadth on disease and target coverage; and length on long-term immune balance. Although promising, the long-term value of CAR-T cell therapy still requires more mature clinical evidence. A multidimensional evaluative framework may help quantify therapeutic effects, capture heterogeneity, and guide more precise treatment strategies. Future studies should refine this framework and determine which patients, targets and products are most likely to provide durable benefit with acceptable risk.

## Outstanding questions

The outstanding questions that future research needs to address include: (1) How should “depth” be standardised and quantified—especially how to correlate peripheral blood indicators with the degree of pathogenic immune memory clearance in lymph nodes/targeted organs? (2) How to balance “breadth” with risks since different types of CAR-T cell therapies have their own disadvantages? (3) How to plan long-term follow-up and delayed toxicity monitoring in advance?

**Search strategy and selection criteria**

Data included in this Personal View was identified by searches of PubMed, Web of Science, and references from relevant articles. The following search terms were used in combination with Boolean operators: (“CAR-T cell” AND “autoimmune disease”) OR (“CD19” AND (“haematological malignancy” OR “haematological autoimmune disease”)) OR (“neuroimmune disorders”). Searches were limited to articles published in English between January 1, 2015, and April 26, 2026. Abstracts and meeting reports were included where necessary to support our statements or views. Studies were excluded if they were not related to CAR-T therapy in autoimmune disease settings, were non-original reviews without relevant data, or lacked sufficient methodological detail.

## Contributors

Q.Y. and J.M. contributed to the conceptualisation of the article. J.S. wrote the article. All authors substantially contributed to the content discussion and reviewed or edited the manuscript before submission. All authors read and approved the final version of the manuscript.

## Declaration of interests

The authors declare no competing interests.
